# Effects of climate changes and road exposure on the rapidly rising legionellosis incidence rates in the United States

**DOI:** 10.1371/journal.pone.0250364

**Published:** 2021-04-22

**Authors:** Xiang Y. Han

**Affiliations:** Department of Laboratory Medicine, The University of Texas MD Anderson Cancer Center, Houston, TX, United States of America; Universite du Quebec a Montreal, CANADA

## Abstract

Legionellosis is an infection acquired through inhalation of aerosols that are contaminated with environmental bacteria *Legionella* spp. The bacteria require warm temperature for proliferation in bodies of water and moist soil. The legionellosis incidence in the United States has been rising rapidly in the past two decades without a clear explanation. In the meantime, the US has recorded consecutive years of above-norm temperature since 1997 and precipitation surplus since 2008. The present study analyzed the legionellosis incidence in the US during the 20-year period of 1999 to 2018 and correlated with concurrent temperature, precipitation, solar ultraviolet B (UVB) radiation, and vehicle mileage data. The age-adjusted legionellosis incidence rates rose exponentially from 0.40/100,000 in 1999 (with 1108 cases) to 2.69/100,000 in 2018 (with 9933 cases) at a calculated annual increase of 110%. In regression analyses, the rise correlated with an increase in vehicle miles driven and with temperature and precipitation levels that have been above the 1901–2000 mean since 1997 and 2008, respectively, suggesting more road exposure to traffic-generated aerosols and promotive effects of anomalous climate. Remarkably, the regressions with cumulative anomalies of temperature and precipitation were robust (R^2^ ≥ 0.9145, *P* ≤ 4.7E-11), implying possible changes to microbial ecology in the terrestrial and aquatic environments. An interactive synergy between annual precipitation and vehicle miles was also found in multiple regressions. Meanwhile, the bactericidal UVB radiation has been decreasing, which also contributed to the rising incidence in an inverse correlation. The 2018 legionellosis incidence peak corresponded to cumulative effects of the climate anomalies, vast vehicle miles (3,240 billion miles, 15904 km per capita), record high precipitation (880.1 mm), near record low UVB radiation (7488 kJ/m^2^), and continued above-norm temperature (11.96°C). These effects were examined and demonstrated in California, Florida, New Jersey, Ohio, and Wisconsin, states that represent diverse incidence rates and climates. The incidence and above-norm temperature both rose most in cold Wisconsin. These results suggest that warming temperature and precipitation surplus have likely elevated the density of *Legionella* bacteria in the environment, and together with road exposure explain the rapidly rising incidence of legionellosis in the United States. These trends are expected to continue, warranting further research and efforts to prevent infection.

## Introduction

Legionellosis is a pulmonary infection caused by the aquatic and soil bacteria *Legionella pneumophila* and other *Legionella* spp. [1, 2]. The infection occurs after inhalation of bacteria-bearing aerosols or soil dust, with an incubation period of several days to 2 weeks [3–5]. Legionellosis impacts public health because it occurs in outbreaks as well as sporadically. While outbreaks can be traced to a single source of exposure, such as aerosols emitted from cooling towers or whirlpool spas [4, 6, 7], the exposure sources for the sporadic cases that account for 75–90% of all legionellosis cases are rarely identified [2, 8–10]. In recent years, the United States has experienced a rapid rise in legionellosis incidence [10], begging an explanation beyond diagnosis, surveillance, and reporting that have changed little in the past 30 years.

Most of the infections occur in warm months [10, 11], reflecting the optimal growth temperature of 25 to 42°C for *Legionella* spp. [2, 12]. Studies have noted the effects of temperature, rainfall, and river systems on legionellosis incidences at various locations [13–16]. We recently showed solar and climate effects on incidence across the United States [11]. The lower incidence rates in the western states corresponded to low precipitation, under an annual threshold of 750 mm, while the higher rates in the eastern states corresponded to more precipitation. Within the eastern states, incidence varied regionally, being promoted by warming temperature but inhibited simultaneously by intensifying solar ultraviolet radiation and longer sunshine hours. These mixed and dynamic effects could be demonstrated in multiple regressions. All these studies together affirm a major role of temperature and rainfall in dictating the abundance of *Legionella* spp. in the environment and shaping legionellosis incidence in concert with human factors.

Viable *Legionella* spp. have been isolated from rainwater puddles on road surfaces, implying potential generation of bacteria-bearing aerosols by motor vehicle traffic and exposure to drivers and passengers [17, 18]. This likelihood is supported by an elevated legionellosis incidence among professional drivers [19–21]. The revelation of inhibitive solar effect, which in converse means lack of inhibition in the absence or attenuation of sunlight, further highlights the significance of road exposure to soil bacteria in the United States, unappreciated previously, in view of daily driving for most Americans [11].

With global warming, the United States has recorded consecutive years of above-norm (1901–2000) annual mean temperatures since 1997 and precipitation surplus since 2008 [22]. In this context, we analyzed the legionellosis incidence rates in the US from 1999 to 2018 for correlation with concurrent temperature, precipitation, solar ultraviolet B (UVB) radiation, and vehicle mileage data in search of an explanation for the rapid rise in incidence. Trend analysis and regression models were applied.

## Materials and methods

This was a correlative study on the surveillance of legionellosis using publicly accessible archived data. We analyzed data for the United States as a whole and for five individual states representing varied climates and legionellosis incidences (California, Florida, New Jersey, Ohio, and Wisconsin).

### Data sources

The legionellosis case data from 1999 to 2018 were from the annual summary of notifiable diseases from the Centers for Disease Control and Prevention (CDC) [23]. The diagnosis required a specific test for *Legionella* spp., such as cultivation, urine antigen test, or strong serologic titer(s), for distinction from diverse etiologic agents of pulmonary infection. These tests had been developed and used since early 1980s, along with reporting to CDC, and from 1980 to 1998, the legionellosis incidence hardly changed in the US [2].

The population data were from the US Census projections [24] using projections 1995 to 2050 for the 1999 population with correction from the 2000 census, projections 2008 and 2009 (averaged) for 2000 to 2011, projection 2012 for 2012 and 2013, projection 2014 for 2014 and 2015, and projection 2017 for 2016 to 2018. Annual incidence rates (all 50 states) in cases per 100,000 population were calculated according to age groups of 0–39 years, 40–64 years, ≥65 years, and all ages. Age-adjusted incidence rates were also used, based on the year 2000 census age brackets, to correct the bias from the aging population.

The temperature and precipitation data for the contiguous United States (since 1895) were from the National Centers for Environmental Information [25]. Annual values of mean temperature and total precipitation as well as seasonal data from May to October were used. Long-term means from 1901 to 2000 were set as benchmark norms to derive yearly or seasonal anomalies during 1999 to 2018; these data were extracted directly from the source. Yearly anomaly values, when summed, yielded cumulative anomalies.

The UVB radiation data were from the UVB Monitoring and Research Program at Colorado State University [26, 27]. UVB data measured wavelengths of 280 to 320 nm, the main germicidal part of sunlight, across the continental United States from coordinates of 24.544°N to 49.344°N in latitude and 66°W to 125.9°W in longitude at a resolution of 0.1°. The average US UVB annual sum, in kilojoules per square meter (kJ/m^2^), represented the mean value from 149,400 coordinates. For the analysis of UVB mean values in five states, the following coordinates were used: 39.244°N to 41.144°N and 74.1°W to 75.2°W for New Jersey; 39.144°N to 41.444°N and 80.5°W to 84.8°W for Ohio; 42.544°N to 46.144°N and 87.6°W to 91.8°W for Wisconsin; 29.844°N to 30.744°N and 82.8°W to 87.5°W for northern Florida and 24.544°N to 30.744°N and 80.4°W to 82.7°W for southern Florida (then combined); and 34.544°N to 41.944°N and 120.4°W to 124.2°W for northern California and 32.744°N to 34.544°N and 114.5°W to 120.4°W for southern California (then combined).

The vehicle mileage data were from the Bureau of Transportation Statistics, including urban, rural, and total vehicle miles [28].

### Statistical analyses

Data handling and analyses used Excel version 2013 [29], including plots for trend, fitting models, and correlations, and linear regressions between various parameters (explanatory variables) and incidence rates (outcome variables). Simple linear regressions were applied to each of the climate and vehicle mileage parameters first, followed by multiple linear regressions with two to all parameters to assess the promotive and/or inhibitive role of each parameter. Cautions were taken during multiple regressions to recognize and avoid spurious results due to data comparability or inherent relatedness, known as multicollinearity [30]. *P* values of 0.05 or less were deemed to be statistically significant.

It was noted that the incidence rates, in cases/100,000 with decimal points, were unsuitable for Poisson regression analysis that requires integers. Instead, they were taken as a normal approximation to Poisson distribution for linear regression analysis, a practice in epidemiology for large scale studies [31], such as the current one. Log transformation of the incidence rates was used at times for optimal fitting. The validities of analysis and assumptions were verified in a statistics package (IBM SPSS version 24) through such tests as Kolmogorov-Smirnov, residual analyses and P-P plots. Although each variable represented a time series from 1999 to 2018, potential cumulative effects and/or interactions of the explanatory variables made the datasets beyond simple time series. Thus, proper interpretation of significant correlations between the incidence rates and explanatory variables and model identification/fitting required biological relevance for support. The primary purpose of model construction was examination of the importance of explanatory variables, which placed focus on multiple regressions for their potential interactions and relative contributions. Autocorrelations were assessed with Durbin-Watson values.

## Results

### Age specific and adjusted incidence trends

**Fig 1:**
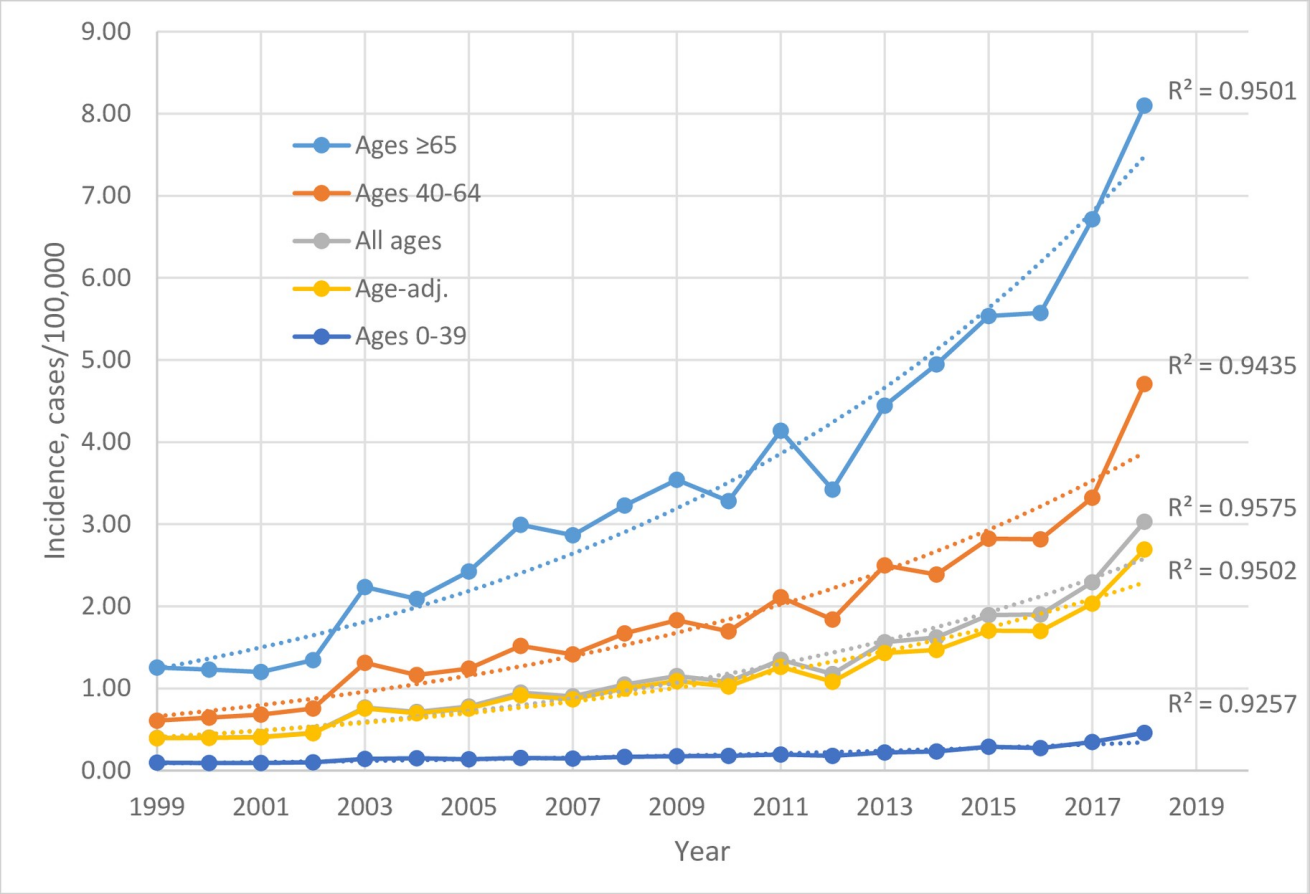
Age-specific, all ages, and age-adjusted legionellosis incidence rates in the United States, 1999–2018, with exponentially fitted dot lines and R^2^ values.

The age-specific, all-age, and age-adjusted legionellosis incidence rates are shown in Fig 1 (Table 1). They remained at base line from 1999 to 2001 and then increased remarkably to reach the peak in 2018, with some year-to-year fluctuations. For the age group of 0–39 years who are least vulnerable to the infection, the rate rose 4.8-fold, from 0.10/100,000 in 1999 to 0.46/100,000 in 2018; for the age group of 40–64 years, the rise was 7.7-fold, from 0.61/100,000 to 4.71/100,000; for the age group of ≥65 years, being most vulnerable, the rise was 6.5-fold, from 1.26/100,000 to 8.1/100,000; and for all ages, the rise was 7.7-fold, from 0.40/100,000 to 3.03/100,000. The age-adjusted rate rose 6.8-fold, from 0.40/100,000 in 1999 (with 1108 cases) to 2.69/100,000 in 2018 (with 9933 cases). Consistent with the aging population, the all-age rates were slightly higher than the age-adjusted ones, in recent years in particular. For example, the age group of ≥65 years constituted 16.0% of the population in 2018, in comparison to 12.4% in 2000.

**Table 1:** Age-specific and age-adjusted legionellosis incidence rates in cases per 100,000 population in the United States, 1999–2018, and data fitting analyses.

Incidence year or parameter	Age group (years)				Age adjusted
	0–39	40–64	≥65	All ages	
**1999**	0.10	0.61	1.26	0.40	0.40
**2000**	0.09	0.64	1.23	0.40	0.40
**2001**	0.09	0.68	1.20	0.41	0.41
**2002**	0.10	0.76	1.34	0.46	0.45
**2003**	0.14	1.31	2.24	0.77	0.75
**2004**	0.15	1.16	2.09	0.72	0.70
**2005**	0.14	1.24	2.42	0.78	0.76
**2006**	0.15	1.52	2.99	0.95	0.92
**2007**	0.15	1.41	2.87	0.90	0.87
**2008**	0.17	1.67	3.23	1.05	1.00
**2009**	0.17	1.83	3.54	1.15	1.09
**2010**	0.18	1.70	3.28	1.08	1.02
**2011**	0.20	2.11	4.14	1.35	1.26
**2012**	0.18	1.84	3.42	1.17	1.08
**2013**	0.22	2.50	4.44	1.57	1.43
**2014**	0.23	2.39	4.95	1.62	1.47
**2015**	0.29	2.82	5.54	1.89	1.71
**2016**	0.28	2.82	5.57	1.90	1.70
**2017**	0.35	3.32	6.71	2.29	2.04
**2018**	0.46	4.71	8.10	3.03	2.69
**Fold increase, 2018 vs. 1999**	4.8	7.7	6.5	7.7	6.8
**R^2^, linear fitting 1999–2016**	0.9076	0.9498	0.9486	0.9497	0.9509
R^2^, linear fitting 1999–2018	0.799	0.8618	0.9102	0.8769	0.8806
**R^2^, exponential fitting 1999–2018**	0.9257	0.9435	0.9501	0.9575	0.9503*
** Coefficient**	e^0.0713^	e^0.093^	e^0.0945^	e^0.0979^	e^0.0912^
** Annual incidence increase**	1.074	1.097	1.099	1.103	1.095

The rate rises were linear from 1999 to 2016, but accelerated in 2017 and 2018, making the 1999–2018 curves fit better exponentially than linearly (Fig 1). From the exponentially fitted coefficients, an annual incidence increase of ~110% was derived (Table 1). These results suggest cumulative effects from strong underlying causes, prompting searches for environmental and human exposure factors.

Potential roles or bias from diagnosis, reporting, and/or surveillance were also assessed. While a direct assessment was difficult, an indirect approach was used via a comparison with the incidence trend of another infection, i.e., invasive pneumococcal infection. This bacterial infection is similar to legionellosis in clinical manifestations (mainly pneumonia), specific diagnostic approaches (mainly cultivation of causative bacteria and urine antigen test), and predilection for the aged (65 years and older), but different in exposure source and route (close contact with an infected person or carrier). These data were available form CDC during 2010 to 2018. As shown in S1 Fig, the incidence rates of invasive pneumococcal infection changed little during the 9 years, suggesting stability in the diagnosis and reporting of pulmonary infections. Therefore, together with the stable legionellosis incidence during 1980–1998 [2], it was unlikely that the diagnosis, surveillance, and/or reporting contributed significantly to the current rapid rise of the incidence.

### Effects of climate changes

**Fig 2:**
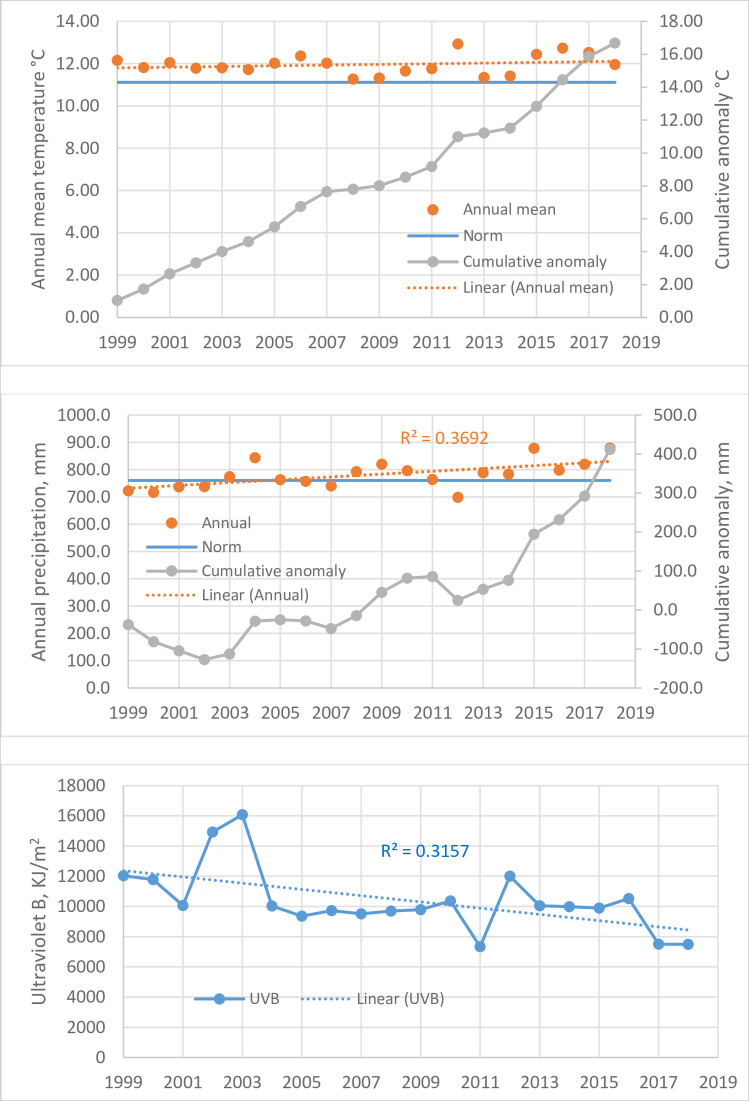
Climate changes in the United States, 1999–2018. Top (A): Annual mean temperature (°C); Middle (B): annual precipitation (mm); Bottom (C): annual sum of solar ultraviolet B radiation (kJ/m^2^).

Changes in temperature, precipitation, and UVB radiation were seen during the 20-year period studied (Fig 2, S1 Table). From 1999 to 2018, the US average annual mean temperatures ranged from 11.27°C to 12.93°C, consistently above the 1901–2000 norm of 11.12°C by 0.15°C to 1.81°C, averaging 0.83°C or 7.5% (Fig 2A). In fact, the anomaly streak started with 11.22°C in 1997 and 12.35°C in 1998 and has continued to 11.49°C in 2019, for a total of 23 years thus far. Cumulative anomalies rose remarkably, from 1.03°C in 1999 to 17.05°C in 2018 (Fig 2A).

The precipitation anomalies were more recent (Fig 2B). During 1999–2007, the annual precipitations fluctuated around the 1901–2000 norm of 760.5 mm. Starting in 2008, 10 of the 11 years showed above-norm precipitation, averaging 802.2 mm. The only exception was 2012, a very dry year (699.3 mm), in which the record (since 1895) high annual mean temperature (12.93°C) also occurred. The anomalies were most pronounced in 2015 (878.6 mm) and in 2018 (880.1 mm), which exceeded the norm by 118.1 mm (15.5%) and 119.6 mm (15.7%), respectively. An upward trending from 1999 to 2018 was significant (R^2^ = 0.3692, *P* = 0.004, Fig 2B). Cumulative anomalies also rose markedly from 2008 to 2018. The anomalous streak continued in 2019 with 883.4 mm, a record (since 1974) level of 16.2% over the norm.

In contrast to the rising temperature and precipitation during the 20 years studied, the UVB radiation trended downward significantly despite considerable fluctuations (R^2^ = 0.3157, *P* = 0.010, Fig 2C). The lowest was in 2011 (7334 kJ/m^2^). The trend continued in 2019 with a level of 8707 kJ/m^2^, below the 1999–2018 mean of 10406 kJ/m^2^.

The climate parameters were analyzed in linear regressions with the age-adjusted legionellosis incidence rates (Table 2). In simple regressions, the annual mean temperature did not correlate with incidence rates, but precipitation showed a positive correlation, suggesting a promotive effect, and UVB radiation showed an inverse correlation, suggesting an inhibitive effect. In multiple regressions, better correlations were obtained, particularly for temperature when combined with precipitation, suggesting a synergistic effect. Because of the seasonality of legionellosis in the summer and fall, the analyses were repeated using the temperature and precipitation data from May to October. The results showed significant correlations with temperature in simple regression (*P* = 0.023) and multiple regressions (*P* = 0.0008) along with precipitation (Table 2).

**Table 2:** Linear regressions between age-adjusted legionellosis incidence rates and concurrent temperature, precipitation, and UVB radiation in the United States, 1999–2018.

Regression/parameter (n = 20)	R^2^	Coefficient	*P* value	Interpretation
**Simple linear with annual data**				
Annual mean temperature	0.0341	0.2369	0.44	No correlation
Annual precipitation	0.4997	0.0084	4.9E-04	Promotive
Annual UVB radiation	0.3304	-0.00016	0.008	Inhibitive
**Multiple linear with annual data**				
Adjusted R^2^	0.5917	--	5.4E-04	Modest correlation
Annual mean temperature	--	0.3472	0.0875	Weakly promotive
Annual precipitation	--	0.0073	0.002	Promotive
Annual UVB radiation	--	-8.3E-05	0.0848	Weakly inhibitive
**With May to October seasonal data**				
Seasonal mean temperature	0.2138	0.7795	0.023	Promotive
Seasonal precipitation	0.3105	0.0096	0.0063	Promotive
Adjusted R^2^, multiple regressions	0.6281	--	8.7E-05	Modest correlation
Seasonal mean temperature	--	0.8778	8.4E-04	Promotive
Seasonal precipitation	--	0.0106	2.6E-04	Promotive
**With cumulative anomaly**				
Cumulative temperature anomaly	0.9145	0.1234	4.7E-11	Strongly promotive
Cumulative precipitation anomaly	0.9148	0.0040	4.6E-11	Strongly promotive
Adjusted R^2^, multiple regressions*	0.9547	--	1.5E-12	Robust correlation
Intercept		0.4816	3.2E-04	
Cumulative temperature anomaly	--	0.0646	4.5E-04	Strongly promotive
Cumulative precipitation anomaly	--	0.0021	4.4E-04	Strongly promotive
Adjusted R^2^, multiple regressions	0.9318	--	4.7E-11	Robust correlation
Intercept		-1.201	0.028	
Cumulative temperature anomaly	--	0.0964	9.2E-07	Strongly promotive
Annual precipitation x total vehicle miles	--	6.5E-07	0.018	Promotive

*A separate regression with 1999–2008 dataset (n = 10) yielded an adjusted R^2^ of 0.8807 and significant *p* values for regression (2.4E-04) and temperature anomaly (3.1E-04) but not for precipitation anomaly (0.42).

Strength of correlation: robust = R^2^ value above 0.7 with p value at E-06 or less; modest = R^2^ value of 0.6 to 0.7 with p value of 0.001 or less.

**Fig 3:**
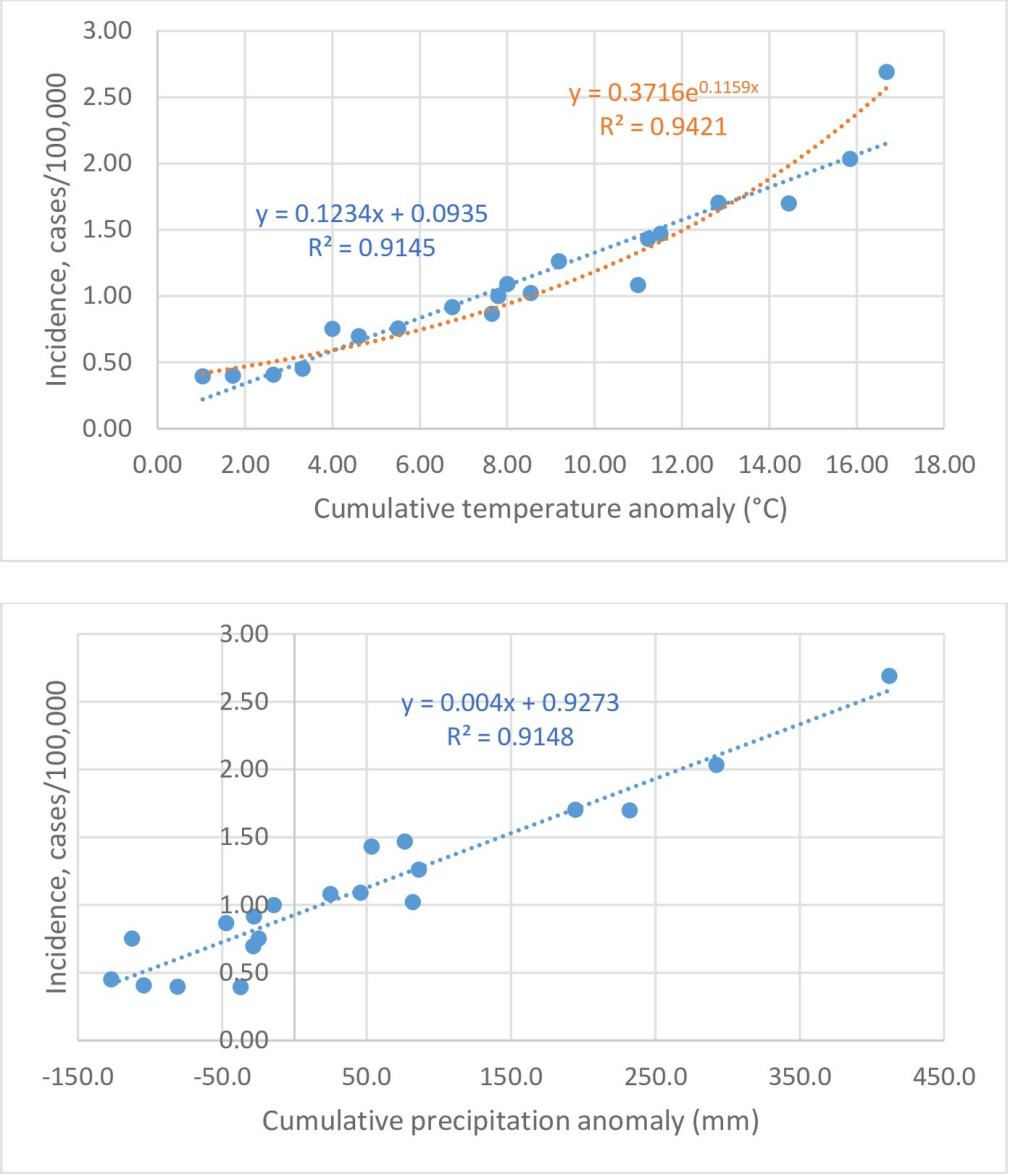
Correlations between age-adjusted legionellosis incidence rates and cumulative anomalies of temperature (up) and precipitation (low) in the United States, 1999–2018, with fitted dot lines, R^2^ values, and equations.

More significantly, the rising cumulative anomalies of temperature and precipitation paralleled the rising legionellosis incidence with robust regressions (Table 2, Fig 3), which explained the cumulative effects and implied possible changes in the ecology of *Legionella* bacteria. The correlation with temperature anomaly also fitted better exponentially than linearly (R^2^ = 0.9421 versus R^2^ = 0.9145, Fig 3). In addition, from 1999 to 2008, prior to the rising trend of precipitation surplus, only temperature anomaly correlated significantly with the rise in incidence, in a separate multiple regression analysis (adjusted R^2^ = 0.8807, *P* = 0.0003) (Table 2 footnote). This result explained the first significant incidence increase in 2003, seven years after the start of anomalous run.

Together, the climate analyses suggest that rising temperature and precipitation and decreasing UBV radiation have contributed to the rising legionellosis incidence. The 2018 incidence peak could be attributed to the cumulative effects, record high precipitation (880.1 mm), near record low UVB radiation (7488 kJ/m^2^), and continued above-norm temperature (11.96°C) (S1 Table). Examples of yearly fluctuations included the 2011 incidence peak (1.26/100,000), corresponding to the record low UVB level (7334 kJ/m^2^), and the 2012 incidence valley (1.08/100,000), corresponding to record drought and a high UVB level (12001 kJ/m^2^) (S1 Table).

### Correlation with vehicle miles driven

**Fig 4:**
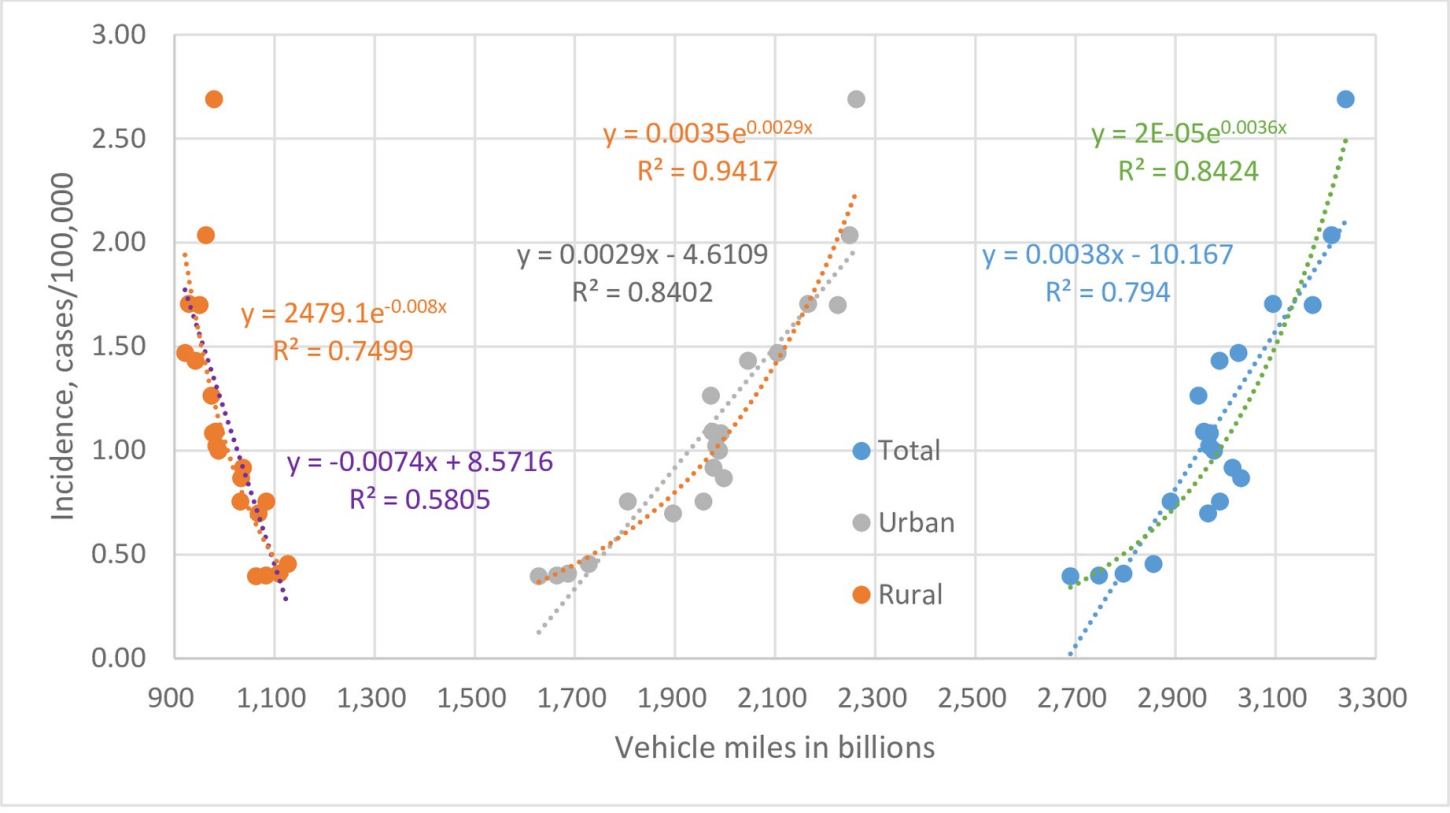
Correlations between age-adjusted legionellosis incidence rates and vehicle miles driven in the United States, 1999–2018, with fitted dot lines, R^2^ values, and equations.

From 1999 to 2018, the vehicle mileage driven in the United States changed gradually with decrease of rural miles and increase of urban miles, resulting in a net increase of total miles (S2 Table). As shown in Fig 4, the urban vehicle miles rose 1.4-fold from 1628 billion miles in 1999 to 2262 billion miles in 2018, and correlated exponentially with the rising legionellosis incidence rates (R^2^ = 0.9417, *P* = 1.5E-12). The total vehicle miles rose 1.2-fold from 2690 billion miles in 1999 to 3240 billion miles in 2018, and also correlated with incidence (R^2^ = 0.8424, *P* = 1.2E-08). The declining rural miles correlated inversely with incidence (R^2^ = 0.7499, *P* = 8.1E-07).

Therefore, the vast total vehicle miles, 9882 miles (15904 km) per capita in 2018, and correlations verified the significance of road exposure to soil bacteria and its contribution to the rise in incidence. In addition, an interactive parameter of total vehicle miles and annual precipitation, in the form of multiplication, was found to promote legionellosis in robust multiple regressions along with cumulative temperature anomalies (Table 2). This result substantiated more road exposure when driving on wet roads during or following precipitation, a biologically sound process that was raised previously [11, 17, 18].

### Analysis of five representative states

**Fig 5:**
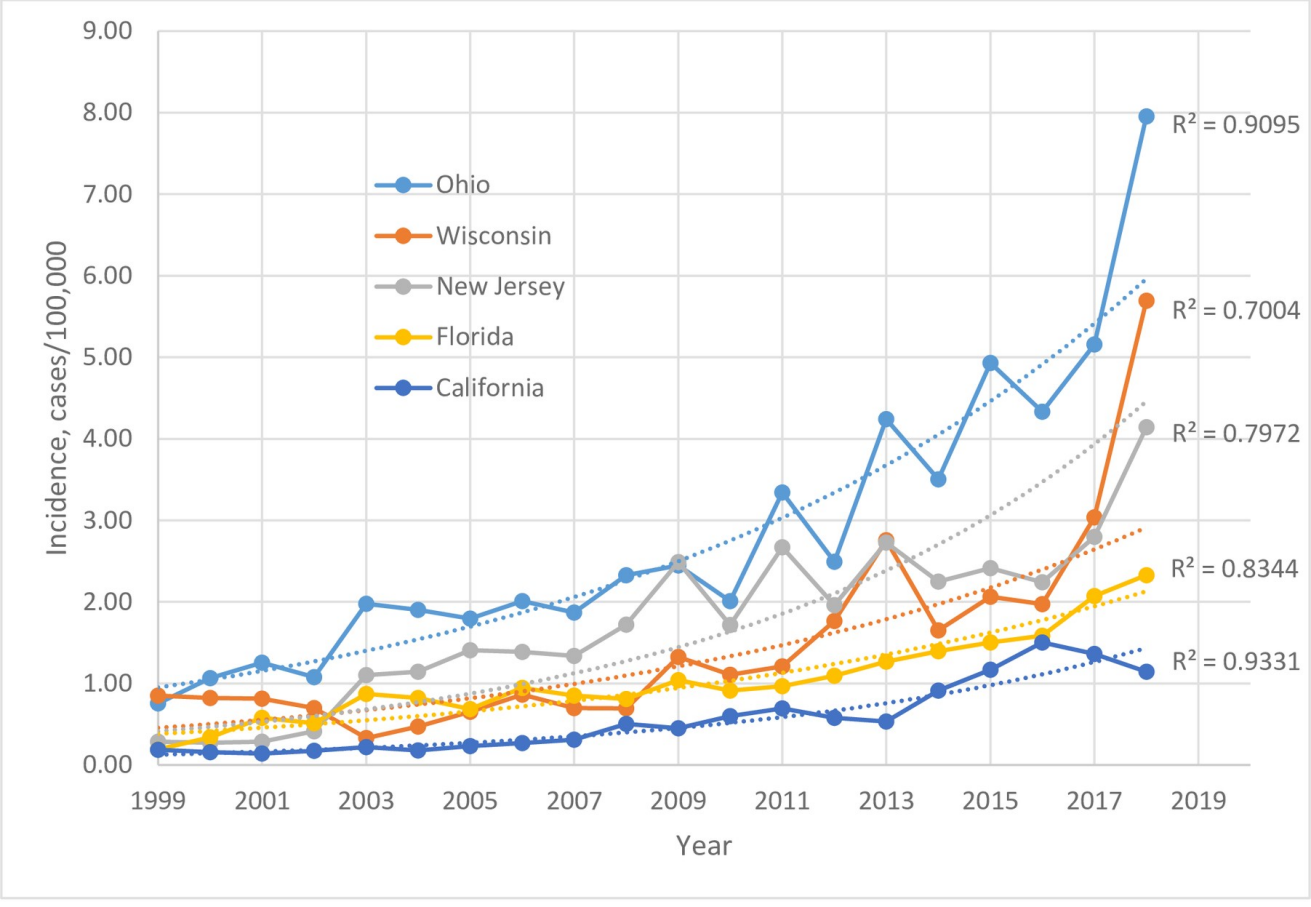
Legionellosis incidence rates (all ages) in California, Florida, New Jersey, Ohio, and Wisconsin, 1999–2018, with exponentially fitted dot lines and R^2^ values.

In view of the diversity of geography and climate across the United States, five representative states were analyzed: California (Pacific coast), Florida (southeast Atlantic coast), Wisconsin (central north), New Jersey (mid-Atlantic coast), and Ohio (Great Lakes region). As shown in Fig 5, these states differed remarkably in legionellosis incidence rates; in 2018, for example, Ohio had the highest all-age incidence (930 cases, 7.96/100,000), Wisconsin (5.69/100,000) and New Jersey (4.14/100,000) were above the US average (3.03/100,000), and California (1.15/100,000) and Florida (2.33/100,000) were below the US average. These states also showed considerable fluctuations in the course of incidence rise during the 20 years.

The regression results for the five states are shown in Table 3 (S3 Table for detailed data). For all states, rising vehicle miles and decreasing UVB radiation levels were significantly correlated to the rising incidence rates. All states showed above-norm temperatures, for all 20 years in California and New Jersey and for 17 to 19 years in the others. An upward rising trend was statistically significant for the warm states of California and Florida but not for the cooler states (S3 Table footnote). The precipitation varied during the 20-year period: dry California experienced an increasing deficit compared with the state norm (average of 7.4% deficit), wet Florida was close to the state norm (average of 1.3% deficit), and the moderate states showed a significant surplus (average of 7.3% to 8.9% surplus).

**Table 3:** Regression analyses between the legionellosis incidence rates and total vehicle miles and climate parameters in selected states, 1999–2018.

Parameter/regression (n = 20)	California	Florida	Wisconsin	Ohio	New Jersey
**Total vehicle miles**					
2018 miles per capita	8,817	10,414	11,333	10,264	8,704
R^2^, simple regression	0.5627	0.8425*	0.4914	0.8108*	0.7653*
*P* value	1.4E-04	1.2E-08	5.8E-04	6.3E-08	4.5E-07
Interpretation	Promotive	Promotive	Promotive	Promotive	Promotive
**Annual UVB radiation**					
R^2^, simple regression	0.2362	0.2426	0.2216	0.2671	0.3265
*P* value	0.030	0.027	0.036	0.020	0.008
Interpretation	Inhibitive	Inhibitive	Inhibitive	Inhibitive	Inhibitive
**Annual mean temperature**					
R^2^, simple regression	0.3864	0.3649	0.0164	0.0122	0.0471
*P* value	0.003	0.005	0.59	0.64	0.36
Interpretation	Promotive	Promotive	NSC	NSC	NSC
**Annual precipitation**					
R^2^, simple regression	0.0089	0.1931	0.3092	0.2387	0.2271
*P* value	0.69	0.053	0.011	0.029	0.034
Interpretation	NSC	Promotive	Promotive	Promotive	Promotive
**Cumulative anomaly**					
R^2^, cumulative temperature anomaly	0.8845	0.9372	0.6791*	0.8894*	0.8317
*P* value	7.2E-10	2.9E-12	7.9E-06	4.8E-10	2.2E-08
Interpretation	Promotive	Promotive	Promotive	Promotive	Promotive
R^2^, cumulative precipitation anomaly	0.7910	0.1065	0.7522	0.927*	0.8728
*P* value	1.6E-07	0.16	7.4E-07	1.1E-11	1.7E-09
Interpretation	Inhibitive	NSC	Promotive	Promotive	Promotive
Adjusted R^2^, multiple regressions	0.8786	0.9450	0.6170	0.9267*	0.9462
*P* value, cumulative temperature anomaly	1.8E-09	7.7E-12	0.007	1.8E-09	1.6E-10
Interpretation	Promotive	Promotive	Promotive	Promotive	Promotive
*P* value, annual precipitation x vehicle miles	0.31	0.045	0.038	0.003	5.3E-06
Interpretation	NSC	Promotive	Promotive	Promotive	Promotive

*Upon logarithmic conversion of the incidence (exponential fitting).

NSC: no significant correlation.

An inhibitive effect is reflected by a negative coefficient.

Due to incidence fluctuations, annual mean temperature and precipitation correlated sub-optimally or poorly in these states, with lower R^2^ values (Table 3). However, cumulative anomalies correlated well, particularly for cooler Wisconsin, Ohio, and New Jersey. The correlations for Ohio also fitted exponentially, suggesting consistent cumulative effects. In multiple regression analyses, the promotive synergism between precipitation and vehicle miles was also noted for the wet states of Wisconsin, Ohio, New Jersey, and Florida, but not for dry California. Conversely, for California, worsening cumulative precipitation deficit correlated inversely with incidence (Table 3), implying an inhibitive effect that opposes the promotive effect of temperature rise. These effects corresponded to the incidence decreases in 2017 and 2018 (Fig 5), unlike the other four states. The record drought in 2013 (since 1895) with a precipitation of 201.4 mm (deficit by 64.6%) also coincided with the incidence drop that year.

The incidence fluctuations in New Jersey and Wisconsin were significant and thus examined further. New Jersey showed fluctuations from 2009 to 2017, averaging 2.36/100,000, before rising again to the peak in 2018 (Fig 5). These variations paralleled precipitation levels, such as those in 2009 (1350.5 mm, surplus by 18.3%), 2011 (1624.3 mm, surplus by 42.2%, a record since 1895), 2016 (1005.6 mm, deficit by 11.9%), and 2018 (1644.9 mm, surplus by 44.0%, a new record) (S3 Table). The correlation accorded with the strong synergy between precipitation and vehicle miles (*P* = 5.3E-06) (Table 3), most notable in New Jersey.

For cold Wisconsin (S3 Table), the incidence rates decreased slightly from 1999 to 2003, rose from 2004 to a peak in 2013 (2.76/100,000), fluctuated in 2014–2016 (averaging 1.89/100,000), and rose again sharply in 2017 to the peak in 2018 (a 3-fold rise in 3 years) (Fig 5). The nadir in 2003 (0.33/100,000) corresponded to the peak UVB radiation activity (15830 kJ/m^2^) and drought, whereas the higher incidence rates in 2013–2018 coincided with a 6-year streak of precipitation surplus (averaging 957.5 mm, surplus by 20.5%). The 2018 peak paralleled record high precipitation (1009.4 mm, surplus by 27.0%), a near record low UVB radiation level (6745 kJ/m^2^), and continued above-norm temperature (6.22°C). More effects of annual temperature variations were also seen. For examples, the 2013 incidence peak followed record (since 1895) heat in 2012 (8.56°C, 48.1% above norm), while the 2014 incidence drop (1.65/100,000) corresponded to the record (since 1973) low temperature that year (4.56°C, 21.1% below norm). The average temperature rise during 1999–2018 was 0.99°C or 17.1% above the state norm of 5.78°C, the highest percentage among the five states. Along with this, the legionellosis incidence also rose most steeply, 17-fold from nadir in 2003 to peak in 2018. Thus, the effect of warming temperature was most pronounced in cold Wisconsin. The dramatic effect undermined regression fitting, in view of the least R^2^ values among the five states (Table 3).

The incidence rates in Ohio were consistently above the national average rates: by 1.9-fold in 1999 (0.75/100,000 vs. 0.40/100,000), 2.1-fold in 2009 (2.45/100,000 vs. 1.15/100,000), 2.6-fold in 2015 (4.93/100,000 vs. 1.89/100,000), and 2.6-fold in 2018 (7.96 vs. 3.03/100,000) (Fig 5). They have been the highest in the United States since 2015, surpassing New York and Pennsylvania that had an incidence rate of 4.39/100,000 and 2.97/100,000 in 2015 respectively. The rates also rose relatively steadily with some year-to-year fluctuations in relation to precipitation surplus or deficit (S3 Table); the curve fitted exponentially with an R^2^ of 0.9095 and a coefficient of e^0.0966^ (Fig 5), the latter equaling an annual increase of 110.1%, identical to the national data. The 2018 peak could be attributed to the cumulative effects, high precipitation (1293.6 mm, surplus by 33.0%), low UVB radiation (6970 kJ/m^2^), and continued temperature anomaly (11.22°C, 9.8% above norm) (S3 Table).

Therefore, in spite of variations, climate changes contributed to the overall rises in legionellosis incidence in the five states with diverse climates. When these states were combined, the incidence rates rose more steadily with little fluctuation, peaking at 2.96/100,000 in 2018 and fitting well exponentially with an R^2^ of 0.9699 and a coefficient of e^0.1017^ (S2 Fig), similar to the all-age incidence curve for the United States. The sharp rises in cooler Wisconsin, Ohio, and New Jersey in 2017 and 2018 suggested an elevation of the density of *Legionella* spp. in the environment as a result of warmer temperature and precipitation surplus.

## Discussion

In this study we have shown that climate changes contributed remarkably to the rapidly rising legionellosis incidence rates in the United States from 1999 to 2018. The incidence rose exponentially at an annual rate of ~110%, higher in recent years, which correlated well with cumulative effects of the above-norm temperature since 1997 and precipitation surplus since 2008. During the 20-year study period, levels of solar UVB radiation, which is germicidal, have been decreasing, correlating inversely with the rise in incidence.

Our data support the notion that the climate anomalies have likely elevated the density of *Legionella* bacteria in the environment, particularly in cooler states. The continued anomalies noted in 2019, a record precipitation of 883.4 mm in particular, forecast a high legionellosis incidence rate (to be available in late 2020). For instance, from January to July (31 weeks), there have been 4522 cases, well above the 2018 number of 4210 cases during the same period (107%) [32]. Our further study on the monthly incidence changes during the 20 years in the U.S. also added filling details on how monthly temperatures, precipitation levels, and sunlight intensity influenced the rise and fall of legionellosis in seasons, to support and complement the above annual data and conclusions [Han XY, unpublished, 2020]. The study showed four main findings: a large seasonal incidence plateau has evolved in recent years from the usually small seasonal incidence hill in the summer and fall 20 years earlier; carryover effect from warm months to cold months was evident; much warmer winters have raised baseline status in incidence and bacterial density in the environment; and strong sunlight in the summer was suppressive whereas weakened sunlight in the fall was permissive.

Climate change affects ecosystems. In a recent experimental study on a soil ecosystem, warming temperature and increased precipitation were found to increase soil temperature and promote the growth of soil microorganisms [33]. Another study showed that warming temperature increased soil microbial activity across a temperature gradient and that the impact of warming was greater in regions with lower temperature [34]. These findings lend support to our epidemiologic evidence of more *Legionella* bacteria in the environment and the result of greater warming effect on legionellosis in cold Wisconsin. The ubiquitous nature of *Legionella* bacteria in soil has been appreciated via microbiological studies [18, 35–37], as well as investigation of soil dust-related outbreaks [5, 38, 39]. Similarly, warming temperature also affects microbes in aquatic environments, such as seasonal variation of *Legionella* spp. in rivers and lakes [40, 41]. Environmental *Legionella* spp. tend to be diverse, but *L*. *pneumophila* serogroup 1 and sequence types are still more common than other species [17, 18, 42]. Among clinical isolates, *L*. *pneumophila* serogroup 1 predominates [43], reflecting higher pathogenicity of this species.

With more *Legionella* bacteria in the environment, more exposures, in dosage and/or frequency, may arise from human activities. The present study has substantiated the role of road exposure to soil bacteria by showing the vast vehicle miles driven in the United States, 3240 billion total miles or 9882 miles (15904 km) per capita in 2018, and the concordant rise with legionellosis incidence during the 20 years studied. Thus, road exposure could be the long-sought hidden source for most sporadic cases, with such exposure conceivably occurring more at night, on days with rain, clouds, fog, or high humidity, driving with open windows or riding a motorcycle, and in northern states with low sunlight intensity. Consecutive rainy days in the summer likely foster the highest levels of road bacteria. Heavy traffic in urban areas, such as during morning and evening rush hours, also aggravates aerosol formation and exposure. This aspect is evidenced by the better correlation between incidence and urban vehicle miles (Fig 4).

Future studies to monitor *Legionella* spp. on roads and in soil are needed to corroborate and further evaluate road exposure risk. In this regard, the current COVID-19 pandemic unfortunately provided an unprecedented opportunity. From the start of lockdowns in mid-March to August 29, 2020, it has drastically reduced the number of legionellosis cases to 40% of otherwise predicted cases following much less driving, which vindicated preliminarily the high significance of road exposure [Han XY, unpublished, 2021].

Earlier studies have shown that higher precipitation levels occurring in warm summer months lead to more legionellosis cases, by a lag of several days to 2 weeks (approximately one incubation period) [13, 14, 44]. Increased precipitation means more water on the ground to aid bacterial proliferation in bodies of water and in soil. More relevantly, precipitation also brings soil bacteria to paved roads, attenuates sunlight to retain bacterial viability, and forms traffic-borne aerosols, all of which favor more and immediate exposure to drivers and passengers in traffic, coinciding the incubation period. This process is now backed by the data on synergistic interaction between precipitation and vehicle miles driven (Tables 2 and 3), most notable in New Jersey, a mid-Atlantic state with record precipitation surpluses in 2011 and 2018, moderate solar strength, annual mean temperature at the US average, and above-norm temperature run since 1979 as recorded [25].

While precipitation promotes road exposure mainly in sporadic cases that account for ~95% of all legionellosis cases in the US in recent decade [Han XY, unpublished, 2021], it may also aid outbreaks in certain settings. For example, in a recent outbreak investigation, heavy rainfalls were noted to precede a fountain-related community outbreak by 1 to 2 weeks to suggest a promotive role [45]. Presumably, in the context of this discussion, the rainfalls could have attenuated sunlight, produced more aerosols, and/or raised atmospheric humidity to aid the survival of aerosols and bacteria for more exposure. High humidity has been shown to promote occurrence of legionellosis in sporadic cases [13, 44]. It is likely that high humidity prolongs traffic-borne aerosols in the air, and thus more host exposure.

The promotive effect of precipitation, shown in this study at the national and state levels, could be viewed as a surrogate of both precipitated water and attenuated sunlight in terms of less ultraviolet radiation and fewer sunshine hours. While the present study analyzed the effect of UVB radiation, the effect of sunshine duration, another significant solar inhibitor of legionellosis [11], was not assessed due to the lack of accurate longitudinal data on hours of sunshine in the United States. This aspect, a limitation, may require further assessment in the future. Lack of precipitation, on the other hand, not only shrinks bodies of water and diminishes soil moisture to limit bacterial proliferation but also reduces road exposure. Accordingly, we observed the 2012 incidence valley in face of a severe drought year in the U.S. (Figs 1 and 2B) as well as the incidence decreases in California in 2017 and 2018 due to sustained drought (Fig 5 and Table 3). These effects were detailed in the seasonality study [Han XY, unpublished, 2020]. These longitudinal results complement our cross-sectional data on the correlation of low legionellosis incidence rates with low precipitation levels in the western U.S. states [11]. Thus, drought suppresses legionellosis.

The significance of road exposure further suggests that many sporadic or community-acquired cases could be prevented through road precautions, such as wearing a mask, driving with closed vehicle windows, and keeping vehicles clean and maintained. These measures are particularly important for individuals with predisposing risk factors, such as weakened immunity, respiratory diseases, diabetes, and old age [1, 6, 46]. Development of a vaccine may be considered as well.

More *Legionella* spp. in the environment, soil in particular due to the ubiquitous nature and lack of purification treatment, may also readily contaminate water supplies, cooling towers, and whirlpool spas, leading to more and/or larger-scale outbreaks. Recent examples include the 2019 outbreak in North Carolina that infected 136 individuals and was related to whirlpool spas [47], the 2015 outbreak in New York City that infected 138 and was related to cooling towers [6], and the 2014–2015 outbreak in Michigan that infected 86 and was related to water supply and hospital settings [48]. These outbreaks typify exposure sources and illustrate challenges to prevent them in the wake of new environmental norms of warmer temperature, precipitation surplus, and more bacteria. They raise the needs of new standards for outbreak surveillance and early detection, maintenance of water-related facilities, and regulations. In fact, the New York City outbreak has led to legislation on the use and maintenance of cooling towers in the city and state [6].

*Legionella-*bearing aerosols emitted from cooling towers may reach individuals on streets and in communities via atmospheric dispersion within a radius. Most reported transmission radii were up to two miles (3.2 km), such as in the New York City outbreak and in another that occurred in Wisconsin [6, 49]. The longest radii reached 11 km and 7.5 km that took place in Quebec City, Canada and in Harnes City in northern France, respectively [50, 51]. The Harnes City outbreak, having occurred in November to January for 79 days, unlike the summer time in the other three outbreaks, conforms lack of solar bactericidal and desiccating effects in the distant drift of aerosols in the winter when the solar strength and temperature in northern France are both at nadir (ultraviolet index of zero to one). The solar strengths in northern United States (New York and Wisconsin) and Canada (Quebec) are moderate in the summer, with peak ultraviolet indices of 6, 5, and 4 in July, respectively, as noted [11, 52], where airborne transmission is more likely at night and early in the morning or in cloudy/rainy days. In a recent analysis of atmospheric conditions during 13 outbreaks that occurred in Europe and were related to cooling towers, foggy weathers were noted to be present in four outbreaks, including the Harnes City outbreak [53]. The water droplets (aerosols) in a fog afloat *Legionella*-laden aerosols from cooling towers longer in the air for dissemination.

Indoor aerosols generated from contaminated whirlpool spas at exhibition, such as in the North Carolina outbreak [47], expose people to the aerobic bacteria readily owing to the ground-level immediately inhalable aerosols, bacterial proliferation promoted by heating and aeration through whirling and bubbling of water, and lack of solar control. A similar whirlpool spas-related outbreak that occurred in the Netherlands earlier led to 188 cases of legionellosis, the largest of its kind [7]. Traffic-borne aerosols, such as generated at night in warm months, share some of these features. These aerosols may expose roadside people, particularly in densely populated areas, in addition to drivers and passenger inside a vehicle. For instance, in New York City, professional drivers and outdoor/street workers in repair, protective services, cleaning, and construction have been noted to incur higher incidence of legionellosis than general working population [20].

Ohio has led the United States in legionellosis incidence for several years, with reasons suggested recently [11]. The present study has further shown contribution from climate changes. The number of 930 cases in a state with 11.7 million residents in 2018 is alarming. The state has the third lowest sunshine hours in the United States, following Alaska and Washington, averaging 2245 hours annually [11], and increased precipitation in 2017 and 2018 further weakened solar inhibition on the incidence. By contrast, California, Florida, New Jersey, and Wisconsin all have longer annual sunshine duration, i.e., 3055, 2927, 2517, and 2468 hours, respectively [11]. These hours correspond inversely to their respective legionellosis incidence.

In conclusion, climate changes and road exposure explain the rapidly rising legionellosis incidence rates in the United States from 1999 to 2018. The incidence is predicted to rise further. Further investigation and preventive actions are urgently needed.

## Supporting information

S1 Fig Age-specific, all ages, and age-adjusted incidence of invasive pneumococcal infection in the United States, 2010–2018. (DOCX)

S2 Fig All age legionellosis incidence rates in the United States and in combined 5 states of California, Florida, New Jersey, Ohio, and Wisconsin, 1999–2018, with exponentially fitted dot lines, R^2^ values, and equations. (DOCX)

S1 Table Temperature, precipitation, and solar ultraviolet B radiation data for the United States, 1999–2018. (DOCX)

S2 Table Vehicle miles driven in the United States, 1999–2018. (DOCX)

S3 Table All-age incidence (cases/100,000), total vehicle miles, temperature, precipitation, and solar ultraviolet B radiation data in selected states, 1999–2018. (DOCX)
