# Supplementary material for: Effects of climate changes and road exposure on the rapidly rising legionellosis incidence rates in the United States
Source: PLoS One. 2021 Apr 22;16(4):e0250364. doi: 10.1371/journal.pone.0250364 (PMC8061983; doi:10.1371/journal.pone.0250364)
Supplement: S1 Fig — (DOCX) [file pone.0250364.s001.docx]

S1 Fig. Age-specific, all ages, and age-adjusted incidence of invasive pneumococcal infection in the United States, 2010-2018.
