# Supplementary material for: Effects of climate changes and road exposure on the rapidly rising legionellosis incidence rates in the United States
Source: PLoS One. 2021 Apr 22;16(4):e0250364. doi: 10.1371/journal.pone.0250364 (PMC8061983; doi:10.1371/journal.pone.0250364)
Supplement: S2 Fig — (DOCX) [file pone.0250364.s002.docx]

S2 Fig. All age legionellosis incidence rates in the United States and in combined 5 states of California, Florida, New Jersey, Ohio, and Wisconsin, 1999-2018, with exponentially fitted dot lines, R^2^ values, and equations.
