# Supplementary material for: Effects of climate changes and road exposure on the rapidly rising legionellosis incidence rates in the United States
Source: PLoS One. 2021 Apr 22;16(4):e0250364. doi: 10.1371/journal.pone.0250364 (PMC8061983; doi:10.1371/journal.pone.0250364)
Supplement: S1 Table — (DOCX) [file pone.0250364.s003.docx]

S1 Table. Temperature, precipitation, and solar ultraviolet B radiation data for the United States, 1999-2018.

| Year | Temperature (°C) | | | | Precipitation (mm) | | | | UVB annual  total (kJ/m^2^) |
| --- | --- | --- | --- | --- | --- | --- | --- | --- | --- |
|  | **Annual** | | **May-October** | | **Annual** | | **May-October** | |  |
|  | **°C** | **Anomaly** | **°C** | **Anomaly** | **mm** | **Anomaly** | **mm** | **Anomaly** |  |
| 1901-2000 norm | 11.12 | 0 | 18.64 | 0 | 760.5 | 0 | 403.4 | 0 | No data |
| 1999 | 12.16 | 1.03 | 18.77 | 0.12 | 723.1 | -37.3 | 385.8 | -17.5 | 12030 |
| 2000 | 11.82 | 0.69 | 19.33 | 0.68 | 716.8 | -43.7 | 374.9 | -28.4 | 11773 |
| 2001 | 12.05 | 0.93 | 19.34 | 0.70 | 737.1 | -23.4 | 394.7 | -8.6 | 10062 |
| 2002 | 11.78 | 0.66 | 19.03 | 0.39 | 737.9 | -22.6 | 404.6 | 1.3 | 14924 |
| 2003 | 11.81 | 0.69 | 19.32 | 0.68 | 775.0 | 14.5 | 420.9 | 17.5 | 16071 |
| 2004 | 11.72 | 0.60 | 18.71 | 0.07 | 844.6 | 84.1 | 481.6 | 78.2 | 10037 |
| 2005 | 12.02 | 0.90 | 19.26 | 0.62 | 764.0 | 3.6 | 403.9 | 0.5 | 9357 |
| 2006 | 12.36 | 1.24 | 19.14 | 0.49 | 757.4 | -3.0 | 401.6 | -1.8 | 9732 |
| 2007 | 12.03 | 0.91 | 19.69 | 1.04 | 741.2 | -19.3 | 407.2 | 3.8 | 9505 |
| 2008 | 11.27 | 0.15 | 18.75 | 0.11 | 793.5 | 33.0 | 424.4 | 21.1 | 9698 |
| 2009 | 11.33 | 0.21 | 18.48 | -0.16 | 820.4 | 59.9 | 480.3 | 77.0 | 9783 |
| 2010 | 11.66 | 0.53 | 19.42 | 0.78 | 796.8 | 36.3 | 441.7 | 38.4 | 10362 |
| 2011 | 11.77 | 0.64 | 19.38 | 0.73 | 764.5 | 4.1 | 391.4 | -11.9 | 7334 |
| 2012 | 12.93 | 1.81 | 19.70 | 1.06 | 699.3 | -61.2 | 363.0 | -40.4 | 12001 |
| 2013 | 11.35 | 0.23 | 19.15 | 0.51 | 788.9 | 28.4 | 452.1 | 48.8 | 10051 |
| 2014 | 11.41 | 0.29 | 19.22 | 0.57 | 783.6 | 23.1 | 438.7 | 35.3 | 9988 |
| 2015 | 12.44 | 1.32 | 19.71 | 1.07 | 878.6 | 118.1 | 473.7 | 70.4 | 9894 |
| 2016 | 12.73 | 1.61 | 19.78 | 1.14 | 798.1 | 37.6 | 433.3 | 30.0 | 10526 |
| 2017 | 12.53 | 1.41 | 19.32 | 0.67 | 820.7 | 60.2 | 445.0 | 41.7 | 7501 |
| 2018 | 11.96 | 0.83 | 19.93 | 1.29 | 880.1 | 119.6 | 482.9 | 79.5 | 7488 |
| 1999-2018 avg | 11.96 | 0.83 | 19.27 | 0.63 | 781.1 | 20.6 | 425.1 | 21.7 | 10406 |
