# Supplementary material for: Effects of climate changes and road exposure on the rapidly rising legionellosis incidence rates in the United States
Source: PLoS One. 2021 Apr 22;16(4):e0250364. doi: 10.1371/journal.pone.0250364 (PMC8061983; doi:10.1371/journal.pone.0250364)
Supplement: S2 Table — (DOCX) [file pone.0250364.s004.docx]

S2 Table. Vehicle miles driven in the United States, 1999-2018.

| Year | Vehicle miles, billions | | |
| --- | --- | --- | --- |
|  | **Urban** | **Rural** | **Total** |
| 1999 | 1628 | 1063 | 2690 |
| 2000 | 1664 | 1083 | 2747 |
| 2001 | 1686 | 1109 | 2796 |
| 2002 | 1728 | 1127 | 2856 |
| 2003 | 1806 | 1084 | 2890 |
| 2004 | 1896 | 1068 | 2965 |
| 2005 | 1957 | 1032 | 2989 |
| 2006 | 1977 | 1037 | 3014 |
| 2007 | 1998 | 1033 | 3031 |
| 2008 | 1988 | 988 | 2977 |
| 2009 | 1975 | 982 | 2957 |
| 2010 | 1982 | 984 | 2967 |
| 2011 | 1972 | 974 | 2946 |
| 2012 | 1992 | 977 | 2969 |
| 2013 | 2046 | 942 | 2988 |
| 2014 | 2105 | 921 | 3026 |
| 2015 | 2166 | 929 | 3095 |
| 2016 | 2225 | 950 | 3174 |
| 2017 | 2249 | 963 | 3212 |
| 2018 | 2262 | 979 | 3240 |
