# Supplementary material for: Effects of climate changes and road exposure on the rapidly rising legionellosis incidence rates in the United States
Source: PLoS One. 2021 Apr 22;16(4):e0250364. doi: 10.1371/journal.pone.0250364 (PMC8061983; doi:10.1371/journal.pone.0250364)
Supplement: S3 Table — (DOCX) [file pone.0250364.s005.docx]

S3 Table. All-age incidence (cases/100,000), total vehicle miles, temperature, precipitation, and solar ultraviolet B radiation data in selected states, 1999-2018.

| State, year | Incidence | Temperature (°C) | | Precipitation (mm) | | Annual UVB  total (kJ/m^2^) | Vehicle miles  (billions) |
| --- | --- | --- | --- | --- | --- | --- | --- |
|  |  | **Annual** | **Anomaly** | **Annual** | **Anomaly** |  |  |
| California |  |  |  |  |  |  |  |
| 1901-2000 norm |  | 14.11 |  | 568.7 |  | No data |  |
| 1999 | 0.19 | 14.44 | 0.33 | 447.8 | -120.9 | 10593 | 300.1 |
| 2000 | 0.16 | 14.89 | 0.78 | 544.3 | -24.4 | 13552 | 306.6 |
| 2001 | 0.14 | 15.06 | 0.95 | 584.7 | 16.0 | 9923 | 310.6 |
| 2002 | 0.17 | 14.89 | 0.78 | 474.5 | -94.2 | 14009 | 321.7 |
| 2003 | 0.22 | 15.22 | 1.11 | 549.4 | -19.3 | 15087 | 323.6 |
| 2004 | 0.18 | 14.94 | 0.83 | 552.7 | -16.0 | 10010 | 328.9 |
| 2005 | 0.23 | 14.78 | 0.67 | 764.5 | 195.8 | 9668 | 329.3 |
| 2006 | 0.27 | 14.78 | 0.67 | 599.7 | 31.0 | 9751 | 327.5 |
| 2007 | 0.31 | 14.94 | 0.83 | 351.3 | -217.4 | 9826 | 328.3 |
| 2008 | 0.51 | 14.94 | 0.83 | 453.4 | -115.3 | 9969 | 327.3 |
| 2009 | 0.45 | 14.89 | 0.78 | 433.1 | -135.6 | 10014 | 324.5 |
| 2010 | 0.60 | 14.28 | 0.17 | 792.0 | 223.3 | 10090 | 322.8 |
| 2011 | 0.70 | 14.11 | 0.00 | 477.3 | -91.4 | 7821 | 320.8 |
| 2012 | 0.58 | 15.28 | 1.17 | 595.6 | 26.9 | 11354 | 326.3 |
| 2013 | 0.53 | 15.17 | 1.06 | 201.4 | -367.3 | 9849 | 329.5 |
| 2014 | 0.91 | 16.33 | 2.22 | 505.5 | -63.2 | 10038 | 332.9 |
| 2015 | 1.17 | 16.00 | 1.89 | 382.0 | -186.7 | 9910 | 335.5 |
| 2016 | 1.50 | 15.61 | 1.50 | 653.5 | 84.8 | 10418 | 340.1 |
| 2017 | 1.36 | 15.72 | 1.61 | 715.5 | 146.8 | 7977 | 343.9 |
| 2018 | 1.15 | 15.61 | 1.50 | 459.5 | -109.2 | 7761 | 348.8 |
| 1999-2018 mean |  | 15.09 | 0.98 | 526.9 | -41.8 | 10381 | 326.4 |
| Florida |  |  |  |  |  |  |  |
| 1901-2000 norm |  | 21.17 |  | 1362.7 |  | No data |  |
| 1999 | 0.19 | 21.78 | 0.61 | 1262.1 | -100.6 | 12607 | 142.0 |
| 2000 | 0.34 | 21.44 | 0.27 | 1023.4 | -339.3 | 10769 | 150.9 |
| 2001 | 0.58 | 21.61 | 0.44 | 1326.1 | -36.6 | 9780 | 170.6 |
| 2002 | 0.51 | 21.83 | 0.66 | 1485.6 | 122.9 | 14633 | 178.4 |
| 2003 | 0.87 | 21.61 | 0.44 | 1492.8 | 130.1 | 14795 | 185.5 |
| 2004 | 0.82 | 21.56 | 0.39 | 1453.4 | 90.7 | 10077 | 196.4 |
| 2005 | 0.68 | 21.56 | 0.39 | 1510.8 | 148.1 | 9373 | 201.5 |
| 2006 | 0.94 | 21.89 | 0.72 | 1041.4 | -321.3 | 9950 | 203.7 |
| 2007 | 0.85 | 22.11 | 0.94 | 1110.5 | -252.2 | 9582 | 206.1 |
| 2008 | 0.81 | 21.56 | 0.39 | 1349.5 | -13.2 | 9821 | 198.6 |
| 2009 | 1.04 | 21.78 | 0.61 | 1365.8 | 3.1 | 9841 | 196.4 |
| 2010 | 0.91 | 20.67 | -0.50 | 1222.2 | -140.5 | 10263 | 195.8 |
| 2011 | 0.97 | 22.06 | 0.89 | 1188.0 | -174.7 | 7568 | 191.9 |
| 2012 | 1.10 | 22.17 | 1.00 | 1399.3 | 36.6 | 11178 | 191.4 |
| 2013 | 1.27 | 22.06 | 0.89 | 1444.5 | 81.8 | 10035 | 192.7 |
| 2014 | 1.40 | 21.44 | 0.27 | 1501.9 | 139.2 | 9939 | 201.0 |
| 2015 | 1.50 | 23.00 | 1.83 | 1342.6 | -20.1 | 10221 | 207.0 |
| 2016 | 1.59 | 22.50 | 1.33 | 1356.6 | -6.1 | 10977 | 215.6 |
| 2017 | 2.07 | 22.67 | 1.50 | 1489.2 | 126.5 | 7958 | 218.8 |
| 2018 | 2.33 | 22.39 | 1.22 | 1537.2 | 174.5 | 8048 | 221.8 |
| 1999-2018 mean |  | 21.88 | 0.71 | 1345.1 | -17.6 | 10371 | 193.3 |
| New Jersey |  |  |  |  |  |  |  |
| 1901-2000 norm |  | 10.78 |  | 1142.0 |  | No data |  |
| 1999 | 0.29 | 12.28 | 1.50 | 1200.4 | 58.4 | 12497 | 65.5 |
| 2000 | 0.27 | 11.22 | 0.44 | 1149.4 | 7.3 | 9492 | 67.4 |
| 2001 | 0.28 | 12.00 | 1.22 | 903.0 | -239.0 | 8986 | 68.7 |
| 2002 | 0.41 | 12.39 | 1.61 | 1166.1 | 24.1 | 13419 | 69.9 |
| 2003 | 1.10 | 11.00 | 0.22 | 1434.6 | 292.6 | 13489 | 69.8 |
| 2004 | 1.14 | 11.67 | 0.89 | 1237.0 | 95.0 | 8955 | 72.8 |
| 2005 | 1.41 | 11.89 | 1.11 | 1239.8 | 97.8 | 8477 | 73.8 |
| 2006 | 1.39 | 12.78 | 2.00 | 1305.8 | 163.8 | 8708 | 75.4 |
| 2007 | 1.34 | 12.00 | 1.22 | 1192.0 | 50.0 | 8621 | 76.2 |
| 2008 | 1.72 | 12.00 | 1.22 | 1199.4 | 57.4 | 8711 | 73.6 |
| 2009 | 2.49 | 11.50 | 0.72 | 1350.5 | 208.5 | 9201 | 73.0 |
| 2010 | 1.72 | 12.61 | 1.83 | 1132.8 | -9.2 | 9539 | 73.0 |
| 2011 | 2.67 | 12.72 | 1.94 | 1624.3 | 482.3 | 6177 | 73.1 |
| 2012 | 1.96 | 13.28 | 2.50 | 1066.5 | -75.5 | 10948 | 74.2 |
| 2013 | 2.73 | 11.78 | 1.00 | 1178.1 | 36.1 | 9424 | 74.5 |
| 2014 | 2.25 | 11.06 | 0.28 | 1243.3 | 101.3 | 9410 | 74.9 |
| 2015 | 2.41 | 12.17 | 1.39 | 1114.0 | -28.0 | 9354 | 75.4 |
| 2016 | 2.24 | 12.72 | 1.94 | 1005.6 | -136.4 | 10344 | 77.1 |
| 2017 | 2.80 | 12.56 | 1.78 | 1141.0 | -1.0 | 6529 | 77.5 |
| 2018 | 4.14 | 12.33 | 1.55 | 1644.9 | 502.9 | 7214 | 77.5 |
| 1999-2018 mean |  | 12.10 | 1.32 | 1226.4 | 84.4 | 9475 | 73.2 |
| Ohio |  |  |  |  |  |  |  |
| 1901-2000 norm |  | 10.22 |  | 972.6 |  | No data |  |
| 1999 | 0.75 | 11.28 | 1.06 | 840.0 | -132.6 | 10507 | 105.5 |
| 2000 | 1.07 | 10.44 | 0.22 | 1037.6 | 65.0 | 9300 | 105.9 |
| 2001 | 1.26 | 11.33 | 1.11 | 947.4 | -25.2 | 9060 | 106.6 |
| 2002 | 1.08 | 11.39 | 1.17 | 980.4 | 7.8 | 13100 | 107.9 |
| 2003 | 1.98 | 10.11 | -0.11 | 1179.1 | 206.5 | 13600 | 108.9 |
| 2004 | 1.90 | 10.72 | 0.50 | 1154.4 | 181.8 | 8880 | 111.7 |
| 2005 | 1.80 | 11.00 | 0.78 | 1018.8 | 46.2 | 8490 | 110.5 |
| 2006 | 2.01 | 11.50 | 1.28 | 1106.4 | 133.8 | 8670 | 111.2 |
| 2007 | 1.87 | 11.28 | 1.06 | 1045.5 | 72.9 | 8630 | 110.6 |
| 2008 | 2.33 | 10.33 | 0.11 | 1120.4 | 147.8 | 8710 | 108.3 |
| 2009 | 2.45 | 10.28 | 0.06 | 927.1 | -45.5 | 8750 | 110.6 |
| 2010 | 2.01 | 10.94 | 0.72 | 933.7 | -38.9 | 9610 | 111.8 |
| 2011 | 3.34 | 11.28 | 1.06 | 1421.1 | 448.5 | 6450 | 112.0 |
| 2012 | 2.49 | 12.22 | 2.00 | 946.9 | -25.7 | 10900 | 112.7 |
| 2013 | 4.24 | 10.50 | 0.28 | 1058.9 | 86.3 | 9170 | 112.8 |
| 2014 | 3.50 | 9.56 | -0.66 | 979.4 | 6.8 | 8890 | 112.8 |
| 2015 | 4.93 | 10.89 | 0.67 | 1092.5 | 119.9 | 8770 | 113.7 |
| 2016 | 4.33 | 12.00 | 1.78 | 949.7 | -22.9 | 9600 | 118.6 |
| 2017 | 5.16 | 11.78 | 1.56 | 1156.0 | 183.4 | 6720 | 119.6 |
| 2018 | 7.96 | 11.22 | 1.00 | 1293.6 | 321.0 | 6970 | 120.6* |
| 1999-2018 mean |  | 11.00 | 0.78 | 1059.4 | 86.8 | 9239 | 111.6 |
| Wisconsin |  |  |  |  |  |  |  |
| 1901-2000 norm |  | 5.78 |  | 794.8 |  | No data |  |
| 1999 | 0.85 | 7.44 | 1.66 | 844.6 | 49.8 | 12363 | 57.0 |
| 2000 | 0.82 | 6.61 | 0.83 | 868.4 | 73.6 | 10838 | 57.3 |
| 2001 | 0.82 | 7.39 | 1.61 | 890.3 | 95.5 | 9353 | 57.3 |
| 2002 | 0.70 | 7.00 | 1.22 | 891.0 | 96.2 | 14130 | 58.7 |
| 2003 | 0.33 | 6.22 | 0.44 | 711.7 | -83.1 | 15830 | 59.6 |
| 2004 | 0.47 | 6.39 | 0.61 | 886.7 | 91.9 | 9422 | 60.4 |
| 2005 | 0.65 | 7.28 | 1.50 | 725.2 | -69.6 | 8632 | 60.0 |
| 2006 | 0.86 | 7.78 | 2.00 | 764.0 | -30.8 | 8906 | 59.4 |
| 2007 | 0.70 | 7.06 | 1.28 | 850.6 | 55.8 | 8855 | 59.5 |
| 2008 | 0.69 | 5.44 | -0.34 | 831.1 | 36.3 | 8979 | 57.5 |
| 2009 | 1.33 | 5.78 | 0.00 | 755.9 | -38.9 | 8823 | 58.2 |
| 2010 | 1.11 | 7.50 | 1.72 | 991.1 | 196.3 | 10121 | 59.4 |
| 2011 | 1.21 | 6.72 | 0.94 | 788.4 | -6.4 | 6694 | 54.4 |
| 2012 | 1.77 | 8.56 | 2.78 | 731.5 | -63.3 | 12407 | 59.1 |
| 2013 | 2.76 | 5.44 | -0.34 | 927.9 | 133.1 | 9633 | 59.5 |
| 2014 | 1.65 | 4.56 | -1.22 | 941.8 | 147.0 | 9220 | 60.1 |
| 2015 | 2.06 | 7.11 | 1.33 | 911.4 | 116.6 | 9026 | 62.1 |
| 2016 | 1.97 | 7.89 | 2.11 | 1000.5 | 205.7 | 9378 | 64.0 |
| 2017 | 3.04 | 7.06 | 1.28 | 954.3 | 159.5 | 6784 | 65.3 |
| 2018 | 5.69 | 6.22 | 0.44 | 1009.4 | 214.6 | 6745 | 65.9 |
| 1999-2018 mean |  | 6.77 | 0.99 | 863.8 | 69.0 | 9807 | 59.7 |

*Estimation based on 2016 and 2017 data due to a change in reporting method in 2018 that resulted in low miles.

Rising trend of annual mean temperature from 1999 to 2018:

California, R^2^ = 0.3314, *P* = 0.008; Florida, R^2^ = 0.3103, *P* = 0.011; New Jersey, R^2^ = 0.1033, *P* = 0.17, not significant; Ohio, R^2^ = 0.0245, *P* = 0.51, not significant; Wisconsin, R^2^ = 0.0170, *P* = 0.58, not significant.
